# Inflammation and lung injury in an ovine model of fluid resuscitated endotoxemic shock

**DOI:** 10.1186/s12931-018-0935-4

**Published:** 2018-11-22

**Authors:** Margaret R. Passmore, Liam Byrne, Nchafatso G. Obonyo, Louise E. See Hoe, Ai-Ching Boon, Sara D. Diab, Kimble R. Dunster, Kavita Bisht, John-Paul Tung, Mohd H. Fauzi, Monica Narula, Sanne E. Pedersen, Arlanna Esguerra-Lallen, Gabriela Simonova, Annette Sultana, Chris M. Anstey, Kiran Shekar, Kathryn Maitland, Jacky Y. Suen, John F. Fraser

**Affiliations:** 1 Critical Care Research Group, Level 3, Clinical Sciences Building, The Prince Charles Hospital, Rode Rd, Brisbane, Australia; 2 University of Queensland, Brisbane, Australia; 3 Australian National University, Canberra, Australia; 4 KEMRI-Wellcome Trust Research Programme, Kilifi, Kenya; 5 Queensland University of Technology, Brisbane, Australia; 6 Research and Development, Australian Red Cross Blood Service, Brisbane, Australia; 7 Department of Emergency Medicine, Universiti Sains Malaysia Health Campus, Kubang Kerian, Kelantan Malaysia; 8 Sunshine Coast University Hospital Intensive Care, Birtinya, Australia; 9 Wellcome Trust Centre for Clinical Tropical Medicine and Department of Paediatrics, Faculty of Medicine, Imperial College, London, UK

**Keywords:** Endotoxemic shock, Inflammation, Lung injury, Matrix metalloproteinases

## Abstract

**Background:**

Sepsis is a multi-system syndrome that remains the leading cause of mortality and critical illness worldwide, with hemodynamic support being one of the cornerstones of the acute management of sepsis. We used an ovine model of endotoxemic shock to determine if 0.9% saline resuscitation contributes to lung inflammation and injury in acute respiratory distress syndrome, which is a common complication of sepsis, and investigated the potential role of matrix metalloproteinases in this process.

**Methods:**

Endotoxemic shock was induced in sheep by administration of an escalating dose of lipopolysaccharide, after which they subsequently received either no fluid bolus resuscitation or a 0.9% saline bolus. Lung tissue, bronchoalveolar fluid (BAL) and plasma were analysed by real-time PCR, ELISA, flow cytometry and immunohistochemical staining to assess inflammatory cells, cytokines, hyaluronan and matrix metalloproteinases.

**Results:**

Endotoxemia was associated with decreased serum albumin and total protein levels, with activated neutrophils, while the glycocalyx glycosaminoglycan hyaluronan was significantly increased in BAL. Quantitative real-time PCR studies showed higher expression of IL-6 and IL-8 with saline resuscitation but no difference in matrix metalloproteinase expression. BAL and tissue homogenate levels of IL-6, IL-8 and IL-1β were elevated.

**Conclusions:**

This data shows that the inflammatory response is enhanced when a host with endotoxemia is resuscitated with saline, with a comparatively higher release of inflammatory cytokines and endothelial/glycocalyx damage, but no change in matrix metalloproteinase levels.

## Introduction

Sepsis represents a serious global health issue, accounting for more than USD $20 billion of total US hospital costs in 2011 [1]. With a current mortality rate of 20–30%, there is an urgent clinical need for improved treatment. Recently reviewed definitions characterize sepsis as life-threatening organ dysfunction due to a dysregulated host response to infection, while septic shock is a subset of sepsis in which particularly profound circulatory, cellular, and metabolic abnormalities substantially increase mortality [2]. Fluid resuscitation remains one of the central interventions in the resuscitation of septic shock, however its safety and efficacy is increasingly being called into question with the only randomized control trial demonstrating harm and a number of observational studies suggesting no benefit with its use [3, 4].

Acute lung injury and acute respiratory distress syndrome (ARDS) are common complications of sepsis. Moreover, sepsis is the most common risk factor for the development of ARDS [5]. Several studies have shown improved clinical outcomes in ARDS patients with the restricted use of intravenous fluid [6–8]. Sepsis is characterized by pulmonary inflammation, neutrophil recruitment, oedema and tissue fibrosis, all leading to decreased pulmonary function [9]. However, it is unclear if the observed incidence of ARDS in sepsis is wholly attributable to the response to infection, or partially due to iatrogenic injury from fluid resuscitation.

Both human sepsis and experimental endotoxemia have been shown to be associated with increases in pro-inflammatory cytokines such as IL-1β and IL-6, with levels predictive of both disease severity and mortality [10, 11]. Activated neutrophils and macrophages, residing in the pulmonary interstitium and alveoli [12], are important in the inflammatory response, secreting serine proteases, cathepsins and metalloproteinases [13]. Alveolar macrophages also produce pro-inflammatory cytokines during tissue injury [14]. Matrix metalloproteinases (MMPs) are a family of zinc-dependent endopeptidases implicated in remodelling of the extracellular matrix (ECM) and the host’s response to pathogen invasion. The activity of MMPs is under the control of pro-inflammatory cytokines and MMP inhibitors, such as tissue inhibitors of matrix metalloproteinases (TIMPs) [15], which then subsequently influence pro-inflammatory cytokine production. These links suggest macrophage function, inflammation and metalloproteinases are closely interconnected. MMP-9, TIMP-2 and TIMP-1 have been shown to be elevated in patients with severe sepsis and septic shock [16, 17] as well as in animal models of endotoxemia [18], while MMP-1 [19], MMP-8 [20, 21] and MMP-13 [22] have also been associated with sepsis.

An ovine model of hyperdynamic endotoxemic shock was recently published in which a traditional resuscitation strategy combining fluid resuscitation and vasopressors was compared to vasopressors alone [23]. Surprisingly, fluid resuscitation appeared to make shock worse with an increased need for vasopressors in the animals receiving fluid resuscitation, with no evidence of improved organ function or metabolism. Indeed, circulating glycocalyx glycosaminoglycan (GAG) hyaluronan, a marker of endothelial damage, was increased. This study aims to investigate if a resuscitation strategy utilizing fluid resuscitation independently contributes to inflammation, lung injury and the development of ARDS in sepsis.

## Methods

### Ovine model of endotoxemic shock

A description of the model and methodology has been published previously [24]. Briefly, hyperdynamic endotoxemic shock was induced by administration of an escalating dose of lipopolysaccharide (LPS; E coli serotype O55:B5; Sigma-Aldrich, St Louis, USA) up to 4 μg/kg/h over 4 h (total LPS dose 11.25 μg/kg). Adequate endotoxemia was confirmed by the occurrence of systemic hypotension with a mean arterial pressure (MAP) less than 60 mmHg after 3 h of endotoxin infusion. In the final hour of endotoxemia animals received either fluid resuscitation with 40 mLs/kg of 0.9% saline over an hour (FR; *n* = 8) or commenced protocolised vasopressor support (NFR; *n* = 8). Noradrenaline was started 60 μg/mL in 5% dextrose (Hospira, Lake Forest, IL, USA) to maintain a MAP between 60 and 65 mmHg. If noradrenaline reached a predetermined 20 μg/min, vasopressin (PPC, Richmond Hill, ON, Canada) was commenced at 0.8 units/h and increased to a maximum of 1.6 units/h if hypotension persisted. The administration protocol was the same for both groups. Post-fluid resuscitation, both groups were monitored for 12 h after the end of endotoxin infusion.

### Sample collection and processing

Bronchoalveolar lavage (BAL) samples were collected from the lower lobe of the left lung at baseline, pre-LPS infusion (or sham) (T1), post-endotoxemia/pre-resuscitation (T2) and 0 and 12 h post-saline resuscitation for ELISA analysis and aliquots cytospun onto slides for inflammatory cell counts. Arterial blood samples were collected for flow cytometry at baseline, T2, 0, 2 and 12 h post-saline resuscitation. Full blood counts were performed using the veterinary mode of the AcT diff™ haematology analyser (Beckman Coulter Australia Pty Ltd., NSW, Australia). Blood films were prepared, stained with Quick Dip (POCD Scientific, NSW, Australia) and used for a manual white blood cell differential count. Neutrophil numbers were calculated based upon the total white cell count from the AcT diff™ and the white blood cell differential count. Tissue samples were removed for histopathology, gene and protein expression studies. Pulmonary oedema was determined by measuring the wet-to-dry-weight of post-mortem left and right upper and lower lobe lung tissue. Serum albumin and total protein were assessed using commercially available kits on the COBAS Integra 400 blood chemistry analyser (Roche Diagnostics, Australia) while total protein was performed on BAL samples using the BCA (bicinchoninic acid) protein assay kit (Pierce Technology, USA).

### ELISA

The concentration of IL-6, IL-1β, IL-8, IL-10 and TNF-α in lung tissue homogenate and BAL fluid was quantified by in-house ELISAs using methods published previously [25–27]. Positive internal controls were used to ensure that inter- and intra- plate variability was < 10%, and confirm the precision and accuracy of all ELISA assays. Hyaluronan was measured in BAL fluid using a standard sandwich ELISA (R&D Systems, Minneapolis, USA).

### RNA extraction and quantitative real-time PCR

Total RNA was isolated from lung tissue using the RNeasy Mini Kit (Qiagen, VIC, Australia). Real-time Quantitative PCR was performed using primers for IL-6, IL-1β, IL-8, TNF-α, IL-10, MMP-1, 2, 8, 9, 13 and TIMP-1, 2, (PrimerDesign, Southampton, UK). The stability of six candidate normalization genes (ACTB, GAPDH, HGPRT, PGK1, PPIA and RPLP0) was evaluated using real-time quantitative PCR. The Qbase PLUS program was used to identify the most stably expressed housekeeping genes and all data was subsequently normalized to a geomean of PGK1 and ACTB (Life Technologies, Grand Island, USA).

### Flow cytometry

EDTA whole blood collected at each time-point was stained with a fluorescein isothiocyanate (FITC) conjugated monoclonal antibody against CD11b (Bio-Rad, CA, USA). Red blood cells were then lysed (FACS-Lyse; BD Biosciences, San Jose, California) and samples were analysed using a FACS Canto flow cytometer (BD Biosciences). Flow cytometry data was analyzed using FlowJo (Tree Star Inc., Ashland, Oregon) with granulocytes identified based upon forward and side scatter gating. The granulocyte CD11b mean fluorescent intensity (MFI) of each sample was determined as a ratio of the MFI of the stained sample to the MFI of its unstained control.

### Histopathology and immunohistochemistry

Portions of left and right lower and upper lobe lung tissue were fixed in 10% buffered formalin for at least 24 h, processed and embedded in paraffin. All sections (5 μm) were stained with haematoxylin and eosin and examined by light microscopy by two independent trained investigators blinded to the slide identity. Pulmonary oedema, vascular and alveolar features, and bronchiole pathology was graded: 0 (normal), 1 (mild), 2 (moderate) and 3 (severe). Features examined included intravascular obstruction, inflammatory cell infiltration, pulmonary congestion, thickening of the alveolar septa, presence of amorphous material and detachment of the bronchiole lining as detailed in Table 1. The upper lobe did not differ significantly from the lower lobe. Lung sections for immunohistochemistry were dewaxed, rehydrated in a series of ethanol and water, then stained with macrophages antibody [MAC 387] (Genetex, CA, USA), MMP-2, MMP-9 (Abcam, Cambridge, UK), TIMP-1 and TIMP-2 (Abbiotec, CA, USA). Primary antibodies were applied and incubated overnight at 4 °C. The sections were then washed and the Vector universal ABC secondary antibody kit (Vector Laboratories, CA, USA) was used to link the primary antibody to the chromogen for 1 h. A positive reaction was detected with 3,3′-diaminobenzidine tetrahydrochloride (Sigma-Aldrich, St Louis, USA), which produces a brown colour at the site of the reaction. Sections were counterstained with haematoxylin, dehydrated in ethanol and xylene and mounted in DePex (ThermoFisher Scientific, Scoresby, Australia). Negative controls were processed using the same methodology, but without the primary antibody. A rabbit IgG polyclonal isotype control (Abcam, Cambridge, UK) was used to ensure staining was not caused by non-specific interactions of immunoglobulin molecules with the sample.

**Table 1:** Lung histology scoring system

Score	Vascular features	Extravascular and alveolar involvement	Bronchiole features
0	Minimal	Minimal	None
1	Blood leaking into interstitium; mild RBC obstruction	Mild inflammatory exudate; areas of patchy oedema with some disordered structure	Mild infiltration of inflammatory cells
2	Mild RBC and vascular obstruction; areas of mild and moderate haemorrhage	Moderate inflammatory exudate; areas of moderate alveolar thickening (25–50% visualized lung)	Moderate infiltration of inflammatory cells; detachment of lining in some bronchioles
3	Diffuse haemorrhage; moderate RBC and vascular obstruction	Moderate-severe inflammatory exudate; Severe alveolar thickening (> 50% visualized lung); loss of structure with amorphous material	Complete loss of bronchiole structure; detachment of lining; cellular debris and inflammatory cell exudate

*RBC* red blood cell

### Quantitative analysis

Images for immunohistochemistry were photographed at a magnification of 250x and visualized using the AxioVision 4.7 Image Analysis system (Carl Zeiss, Germany). Semi quantitative analysis of macrophage number was performed on 20 fields of photographed images.

### Statistics

Data are presented as mean ± SEM. Lung wet-to-dry weights, serum albumin, serum total protein were normally distributed and analysed initially using a two sample, two-tailed t-test assuming equal variances. All other variables were not normally distributed and compared using the Wilcoxon rank-sum test. A multivariable mixed effects linear regression model was used for serum albumin, serum total protein, absolute polymorphonuclear leukocyte (PMN) counts, whole blood CD11b, cytokines and hyaluronan over time to identify differences between groups. All analyses took into account potential confounders including weight of sheep at baseline and fluid balance. Statistical analyses were performed using STATA™ (StataCorp, TX, USA) statistical software package (version 13). A *p* value ≤0.05 was considered significant.

## Results

### Fluid resuscitation increased lung oedema

**Fig. 1:**
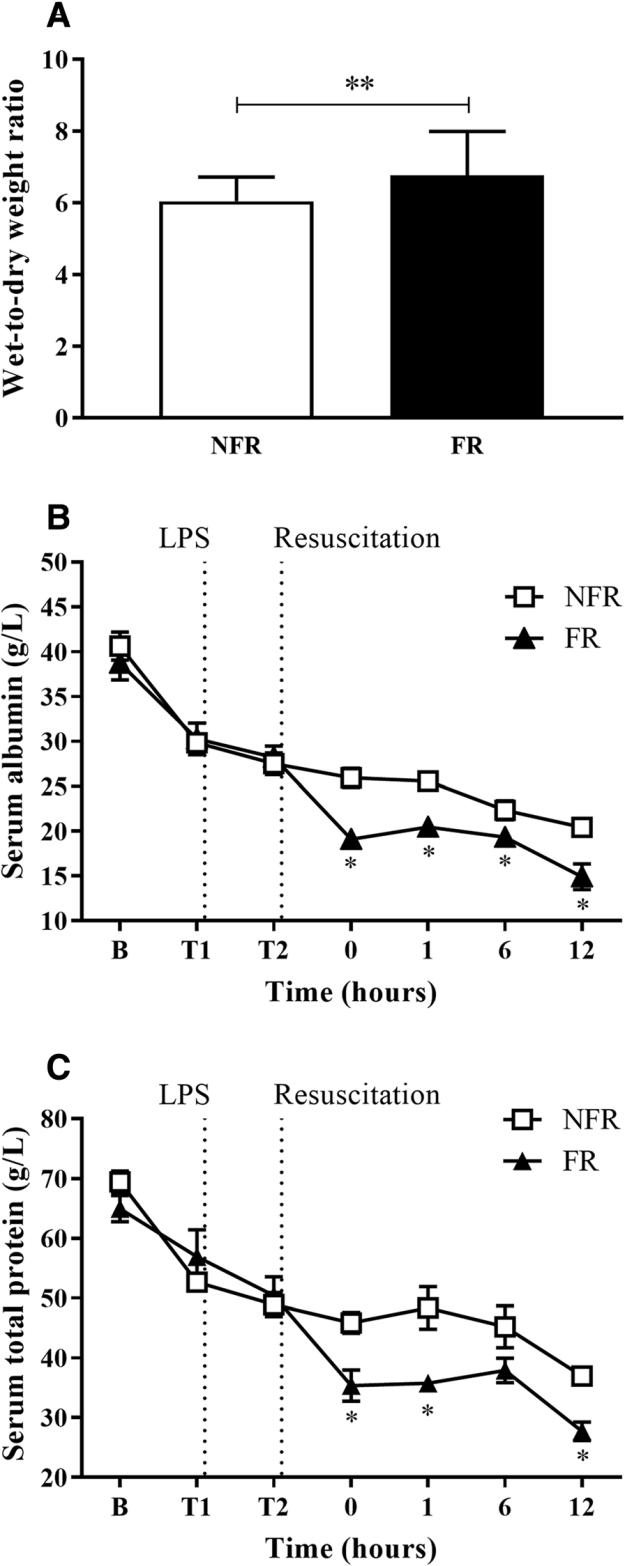
Effect of fluid resuscitation on lung wet-to-dry weight ratios, serum albumin and serum total protein levels. Fluid resuscitation resulted in a significant increase in lung fluid compared to no fluid resuscitation **a** while serum albumin **b** and serum total protein levels **c** decreased throughout the experiment. Data are presented as mean ± SEM. *n* = 8 no fluid resuscitation, *n* = 8 fluid resuscitation. B = baseline; FR = fluid resuscitation; LPS = lipopolysaccharide; NFR = no fluid resuscitation. *p < 0.05, **p = 0.005

Lung wet-to-dry weight ratio was used as a measure of increased capillary permeability and extravascular lung fluid. There was no significant difference between the left and right lobe of lung tissue. FR resulted in a significant increase in lung fluid compared to NFR (Fig. 1a, *p* = 0.005). Serum levels of albumin and total protein (Fig. 1b and c) decreased throughout the experiment with significantly lower levels in the FR group compared to NFR from T0 onwards (*p* < 0.05 and *p* < 0.01 respectively). BAL total protein levels were unchanged.

### Tissue homogenate and BAL levels of IL-6, IL-8, IL-1β and hyaluronan increased with fluid resuscitation

**Fig. 2:**
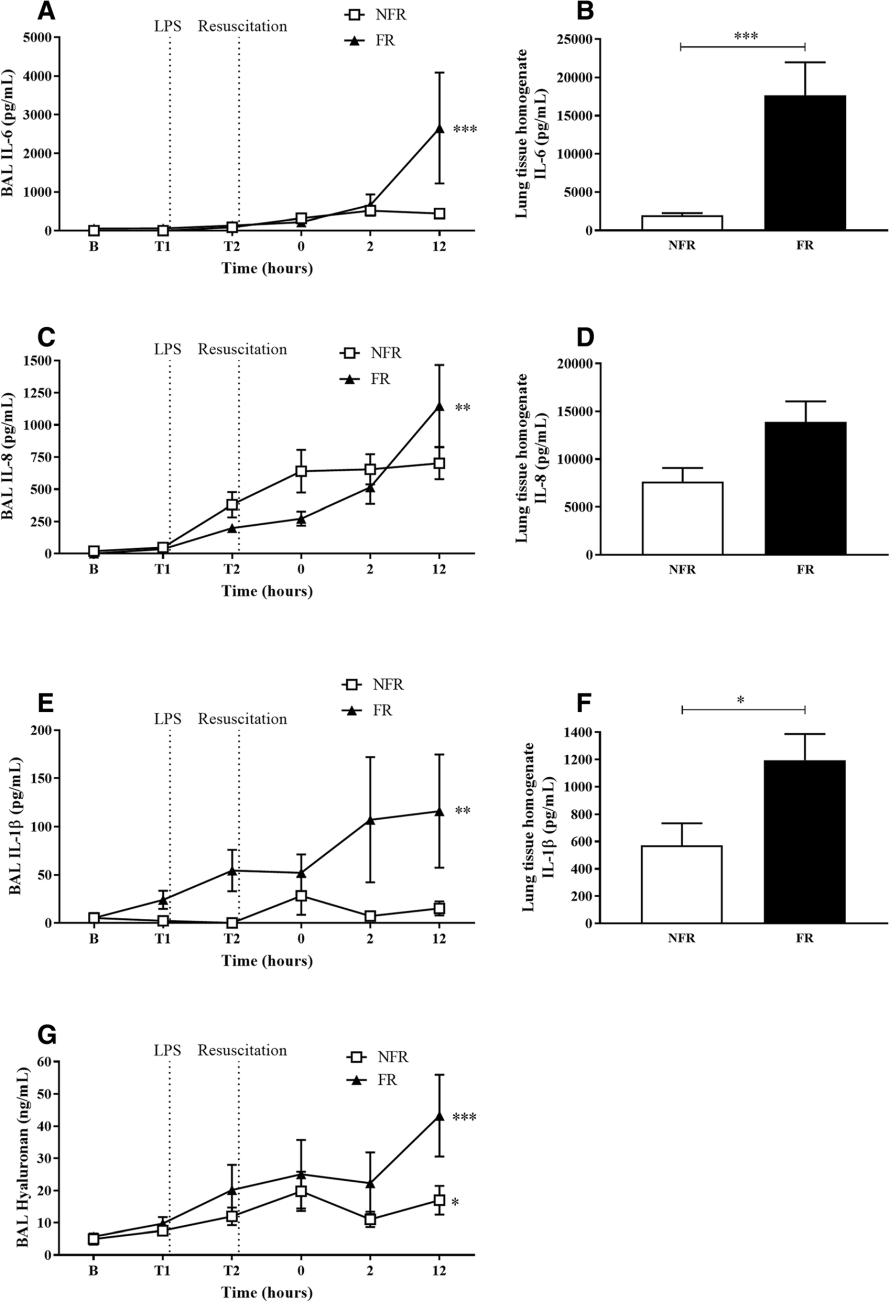
Cytokine and hyaluronan levels in BAL fluid and lung tissue homogenate. Fluid resuscitation significantly elevated levels of IL-6 from baseline to 12 h in BAL (**a**) while IL-6 lung homogenate (**b**) levels were increased 12 h post-saline resuscitation. Levels of IL-8 were also elevated in BAL from baseline to 12 h (**c**) while there was an increased trend in lung homogenate (**d**). IL-1β levels significantly increased in both BAL (**e**) and lung homogenate (**f**) with fluid resuscitation. Hyaluronan levels increased significantly from baseline to 12 h in BAL (**g**) both with and without fluid resuscitation. Data are presented as mean ± SEM. *n* = 8 no fluid resuscitation, *n* = 8 fluid resuscitation. B = baseline; FR = fluid resuscitation; NFR = no fluid resuscitation *p < 0.05, **p < 0.01, ***p < 0.001

Linear regression analysis demonstrated that levels of IL-6 BAL increased significantly from baseline to 12 h in the FR group (Fig. 2a, *p* = 0.002). Levels of IL-6 in the tissue homogenate (Fig. 2b) were significantly elevated after 12 h compared to NFR (*p* < 0.001). IL-8 levels in BAL fluid of the FR group rose significantly from baseline to 12 h (Fig. 2c, *p* = 0.007) while there was a trend for increased expression in the tissue homogenate (Fig. 2d, *p* = 0.06). IL-1β levels in BAL (Fig. 2e) and tissue homogenate (Fig. 2f) were significantly increased in FR compared to NFR (*p* < 0.01 and *p* < 0.05 respectively). From baseline to 12 h glycocalyx GAG hyaluronan levels were also significantly elevated in BAL (Fig. 2g) with NFR (*p* = 0.04) and FR (*p* < 0.001). Although there was no significant difference at 12 h there was a trend for increased levels in the FR group (*p* = 0.09). There was no significant difference in IL-10 or TNF-α levels in either tissue homogenate or BAL.

### Changes in inflammatory and fibrosis markers in the lung with fluid resuscitation

**Fig. 3:**
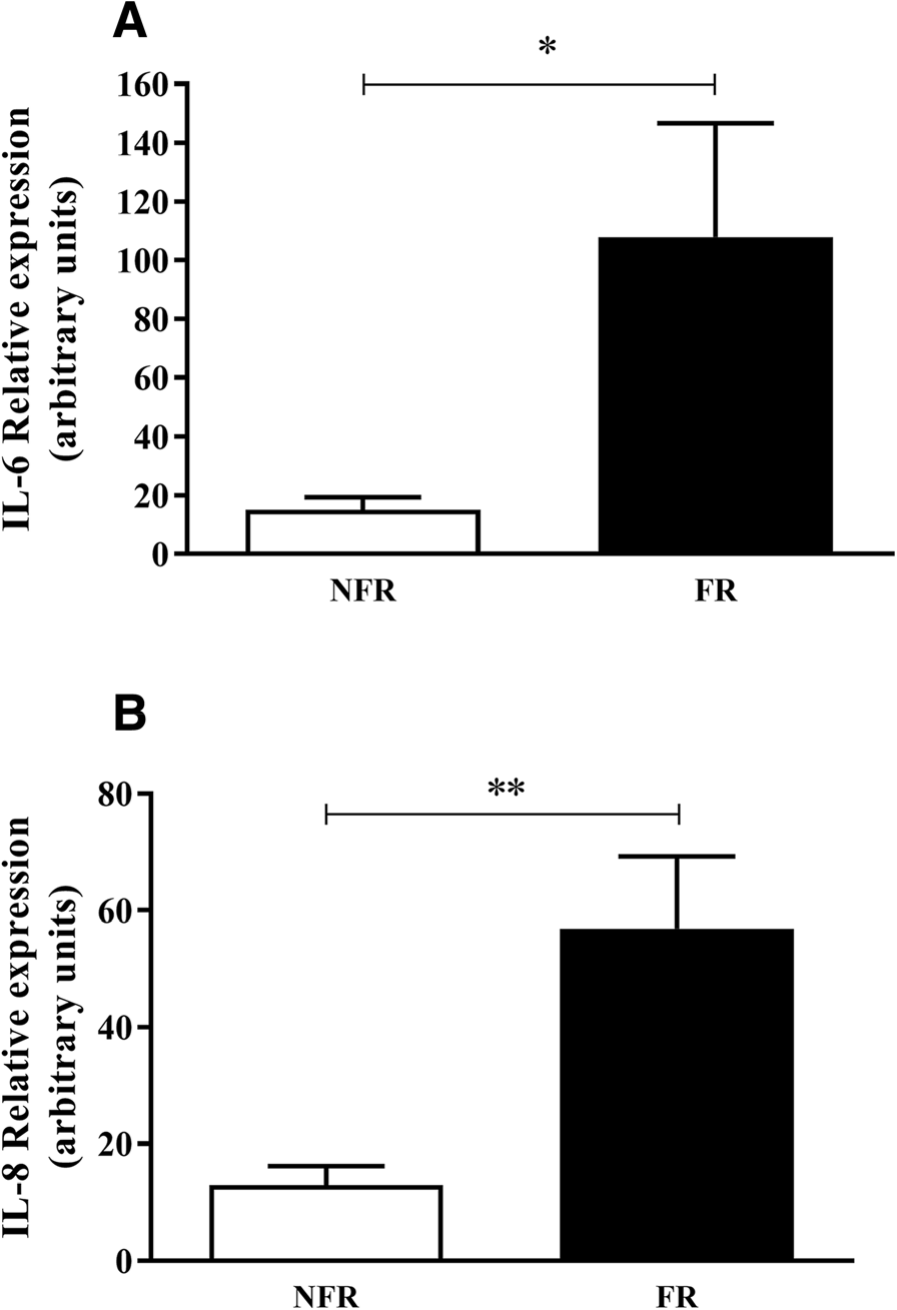
Relative gene expression levels in lung tissue. Levels of IL-6 (**a**) and IL-8 (**b**) were increased with fluid resuscitation. Data are presented as mean ± SEM. *n* = 8 no fluid resuscitation, *n* = 8 fluid resuscitation. FR = fluid resuscitation; NFR = no fluid resuscitation *p < 0.05, **p < 0.01

Gene expression levels of pro- and anti-inflammatory cytokines and extracellular matrix proteins were measured in post-mortem lung tissue. Quantitative real-time PCR showed significant increases in IL-6 (Fig. 3a, *p* = 0.03) and IL-8 (Fig. 3b, *p* = 0.01) with fluid resuscitation. There was no change in the levels of IL-1β, TNF-α, MMP-1, MMP-2, MMP-9, TIMP-1 or TIMP-2. Gene expression levels of IL-10, MMP-8 and MMP-13 were below the limits of detection.

### Endotoxemia changes the percentage of inflammatory cells

**Fig. 4:**
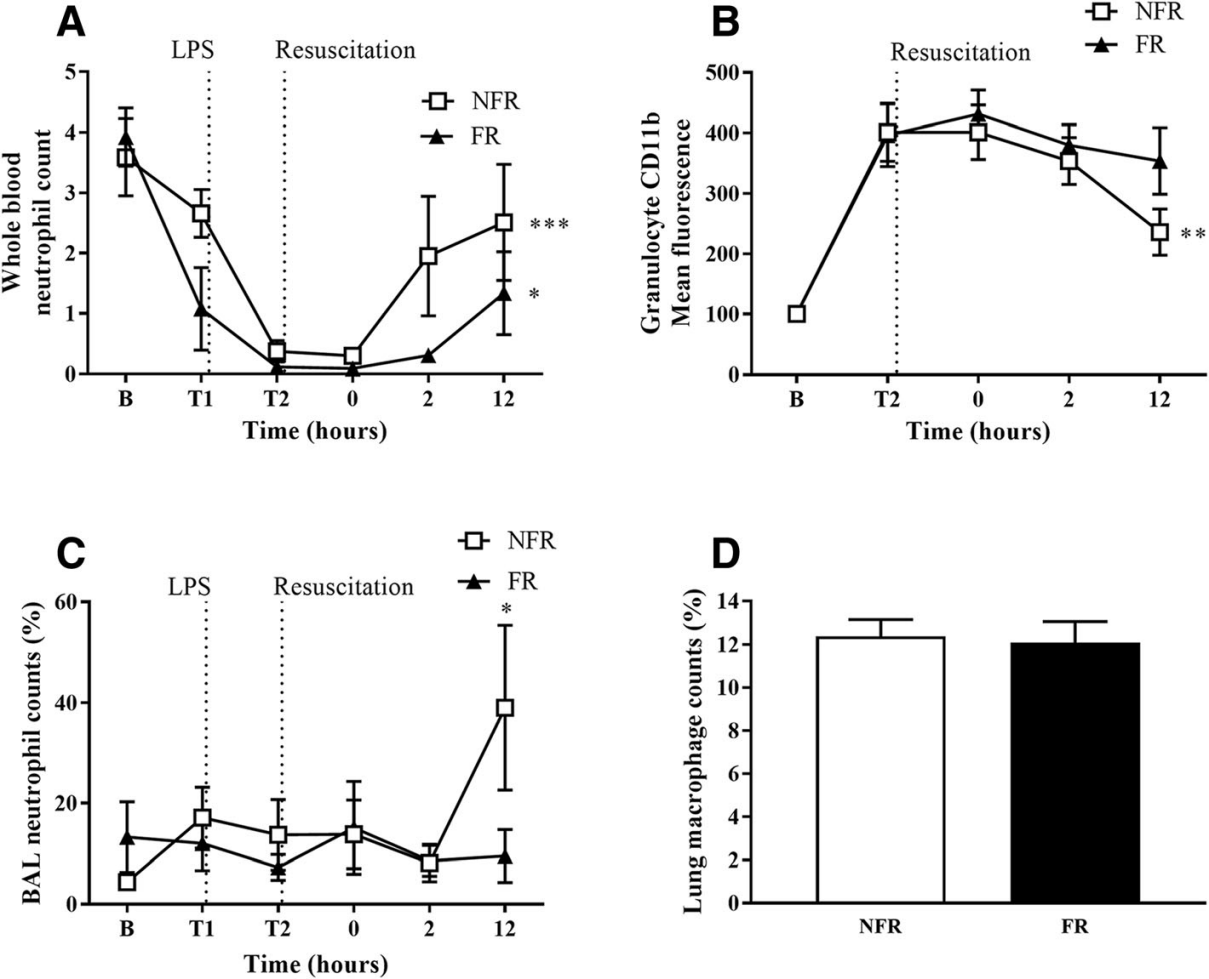
Changes in the number of inflammatory cells with fluid resuscitation. The number of circulating neutrophils decreased with endotoxemia before recovering post-saline resuscitation (**a**) with the NFR group recovering more quickly. Flow cytometry data revealed the levels of CD11b were upregulated on granulocytes in both groups, significantly decreasing post resuscitation in the NFR group (**b**). Neutrophil counts in BAL cytospins, as a percentage of total cells, were increased at 12 h with NFR (**c**). There was no change in the infiltration of macrophages in lung tissue with either group (**d**). Data are presented as mean ± SEM. *n* = 8 no fluid resuscitation, *n* = 8 fluid resuscitation. B = baseline; FR = fluid resuscitation; LPS = lipopolysaccharide; NFR = no fluid resuscitation; PMN = polymorphonuclear leukocyte. *p < 0.05, **p < 0.01, ***p < 0.001

The number of circulating neutrophils in whole blood, as calculated from blood smear analysis and total white cell count, decreased significantly in both groups between baseline and T0 before recovering post resuscitation (Fig. 4a, *p* < 0.05) with the NFR group recovering more quickly. Flow cytometry data revealed the levels of the neutrophil activation marker CD11b increased significantly in both groups pre-resuscitation (Fig. 4b, *p* < 0.001). In the post-resuscitation period CD11b levels fell significantly with NFR (*p* = 0.007) but not in the FR group. Neutrophil counts in BAL cytospins, as a percentage of total cells, were increased at 12 h with NFR (Fig. 4c, *p* = 0.02). There were no significant differences in BAL macrophages, lymphocytes or eosinophils between the two groups. Additionally, immunohistochemical staining was used to stain lung sections with a macrophages antibody (Fig. 4d). There was no significant difference between NFR (12.4 ± 0.78%) and FR (12.1 ± 0.97%).

### Histopathological changes in pulmonary injury with bolus infusion of 0.9% saline

**Fig. 5:**
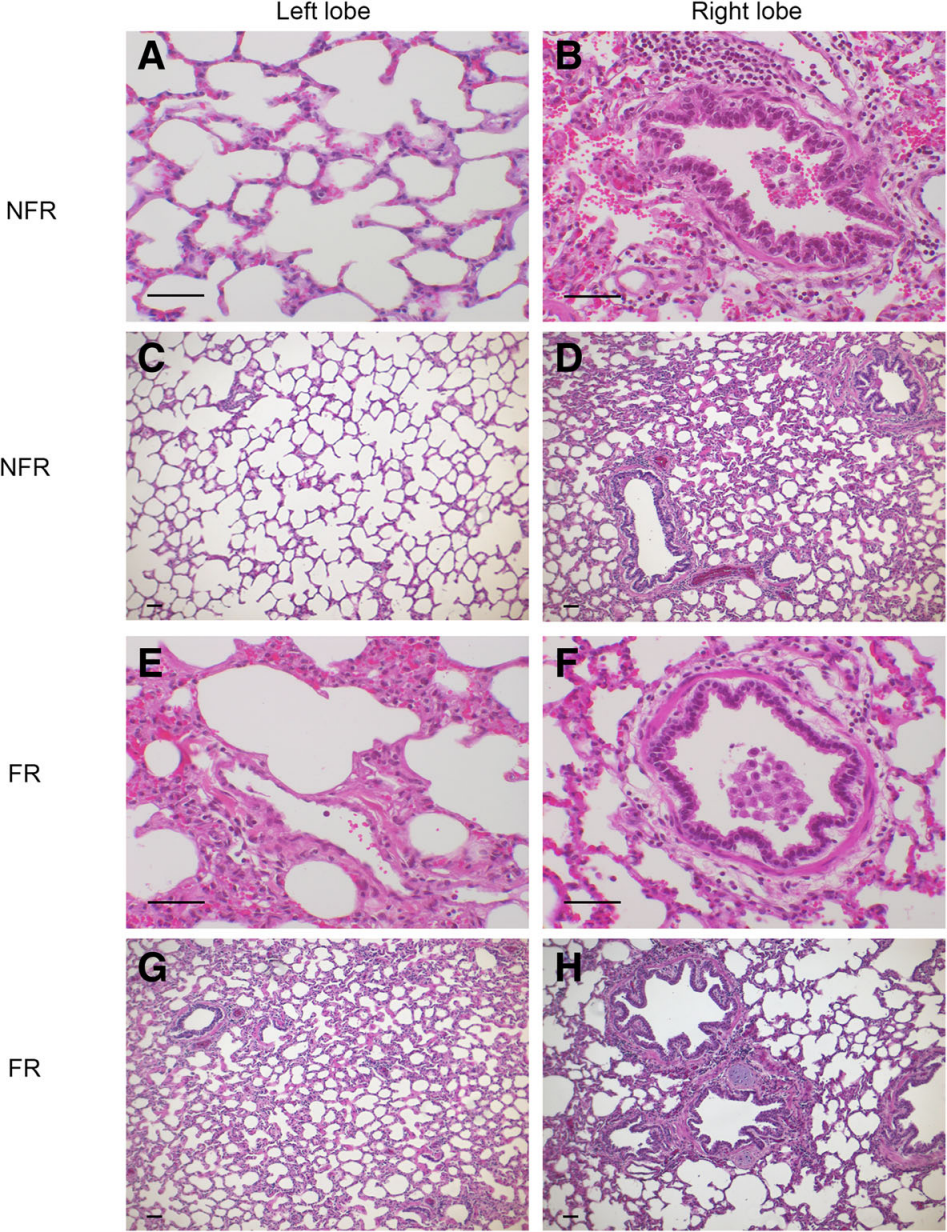
Representative histology showing endotoxemia and saline resuscitation associated changes to bronchial and alveolar histology. The left lobe from NFR (**a**, **c**) and FR (**e**, **g**) groups had minimal inflammatory infiltrate or oedema. Right lobe tissue in NFR (**b**, **d**) and FR (**f**, **h**) groups had some additional damage to bronchioles. FR = fluid resuscitation; NFR = no fluid resuscitation. Scale bars = 50 μm

The left lobe from NFR (1.1 ± 0.4, Fig. 5a, c) and FR (1.2 ± 0.3, Fig. 5e, g) groups had little inflammatory infiltrate or oedema. Right lobe tissue in the NFR (1.7 ± 0.4, Fig. 5b, d) and FR (1.7 ± 0.4, Fig. 5f, h) groups had some additional damage to bronchioles with detachment of the cell lining but this was minimal and did not affect overall scoring. Immunohistochemical staining in the lung also revealed there was no change in secretion of MMP-2, MMP-9 or their inhibitors TIMP-1 and TIMP-2 with saline resuscitation.

## Discussion

Sepsis is the most common pre-disposing condition for the development of ARDS [5], however it remains unclear if this is due to the underlying infection or if resuscitation therapies play a role in the development of lung injury and inflammation. Increasingly it is understood that fluid resuscitation has a wide range of effects beyond merely increasing cardiac output. Previously, in this model we demonstrated that fluid resuscitation resulted in a paradoxical increased need for vasopressor support and increased levels of plasma atrial natriuretic peptide (ANP), troponin and glycocalyx breakdown products (hyaluronan) [23]. Using the same model, we have compared two different resuscitation strategies to determine if there are important differences in pulmonary inflammation due to a resuscitation strategy utilizing fluid bolus therapy. It is important to highlight that animals receiving fluid resuscitation also received more vasopressor support. As a result, the differences observed are between a resuscitation therapy either with or without fluid bolus therapy. This study found that a resuscitation strategy including bolus crystalloids resulted in increased levels of intra-pulmonary cytokines, with a trend for increased BAL hyaluronan, but no change in matrix metalloproteinase levels.

As expected, saline resuscitation resulted in increased extravascular lung fluid, as measured by wet-to-dry weight ratios. Additionally, serum albumin and total protein were significantly lower in the group receiving fluid resuscitation. One possible explanation being increased vascular permeability and albumin loss into the interstitium. This hypothesis is supported by previous work by our group and others demonstrating fluid resuscitation and hypervolemia damages the glycocalyx, a thin gel-like structure lining the vascular endothelium, responsible for maintaining the barrier function of the vascular endothelium [23]. Hypoalbuminemia has been shown to be correlated with sepsis progression [28]. Surprisingly there was no change in the levels of BAL total protein, however extending the study duration beyond 12 h could further elucidate changes in alveolar-capillary membrane permeability. Histological examination of lung tissue in both groups showed damage to bronchioles with detachment of the cell lining and focal areas of alveolar oedema, but this was minimal and did not affect overall scoring. Though the pulmonary damage and oedema resulting from fluid administration was not significant in this series, an extended duration, as often occurs in the clinical situation, may exacerbate these changes.

Cytokines are influential mediators of immune/inflammatory reactions like sepsis. We observed significantly elevated levels of IL-6, IL-8 and IL-1β in lung homogenate and BAL fluid in FR animals compared to the NFR group. This was also associated with increased gene expression of both IL-6 and IL-8 in the lung homogenates of FR animals. These findings are interesting as they suggest increased levels of in-situ inflammatory signalling in the lungs of fluid resuscitated animals. Previously, we measured the levels of circulating cytokines in this model and found remarkably similar levels between the two groups. Taken together, these results suggest that there may be important differences between circulating levels of inflammatory mediators and local levels at the site of injury and inflammation within tissues. While the levels of IL-1β were increased in BAL and tissue homogenate there was no change in transcription. This may be dependent on post-translational modification of the molecule, changes in protein degradation regulation or potential release from pre-formed stores. Though primarily secreted by monocytes and macrophages, Il-1β is known to be produced by neutrophils, dendritic cells, mast cells, endothelial and epithelial cells [29]. In addition to chemotaxis and activation of PMNs, pro-inflammatory cytokines are known to induce structural changes in the glycocalyx [30] and cytokines can stimulate heparanase secretion in human vascular endothelial cells thereby augmenting glycocalyx shedding [31, 32] . These findings are in accordance with previous observations of increased IL-6 and IL-8 in the BAL fluid of patients suffering from ARDS [33]. These cytokines and chemokines may play a role in establishing inflammation in the lungs and the progression to ARDS.

Endotoxin infusion significantly reduced circulating neutrophils in both groups. We and others have previously demonstrated in animal models that this endotoxemia-induced neutropenia is associated with pulmonary and hepatic accumulation of neutrophils [34–37]. In our study, up-regulation of the neutrophil activation marker CD11b was also evident in both groups, corresponding to previous reports of up-regulation of CD11b/CD18 integrin (Mac-1) [38, 39]. Importantly there was not a detectable difference between either the circulating or intra-pulmonary levels of PMNs, however, the percentage of neutrophils in BAL fluid was increased in the NFR group suggesting recruitment to sites of injury. Future research could focus on assessing the correlation between fluid resuscitation and alveolar neutrophil numbers.

MMP’s and their inhibitors, TIMP’s, play a pivotal role in tissue remodelling and are therefore particularly important in diseases such as sepsis, which involve extracellular matrix disruption. MMP-1 is released by endothelial cells under stress conditions leading to subsequent loss of endothelial integrity and increased vessel permeability [40]. Increased levels of active MMP-1 have been associated with lower rates of survival in patients with sepsis, while mouse models show MMP-1 inhibitors prevent sepsis- induced lethality [19]. Our study did not find any significant differences between groups in the levels of either MMP-1 or the inhibitors TIMP-1 and TIMP-2. Both MMP-1 and TIMP-1 were (non-significantly) higher in the NFR animals to a similar degree. These findings are in keeping with the histology results that did not demonstrate significant differences between the two treatment strategies. In contrast to previous studies, [17, 41, 42] there was no change in the levels of TNF-α, MMP-2 and MMP-9 in any of the treatment groups, and the gene expression levels of IL-10, MMP-8 and MMP-13 were below the limits of detection. Our group also investigated the levels of pro- and active MMP-2 and -9 in BAL fluid by zymography (data not shown), but did not find any differences in enzymatic activity between any of the groups. These results suggest a fluid resuscitation strategy does not significantly alter MMP expression in the lung during the early stages of endotoxemia. Longer duration studies would be needed to characterize if there are further changes beyond 12 h as changes in expression levels may not be evident this early in the model.

## Conclusion

In summary, our ovine model of hyperdynamic endotoxemic shock provides promising insights into the lung specific effects of fluid resuscitation. Bolus fluid resuscitation resulted in increased lung oedema and both increased cytokine gene expression and inflammatory signalling within the lung. However, this did not translate into increased MMP expression or histological injury. Further studies are required to understand if the cytokine changes observed result in delayed progression of lung injury and ARDS.

## Abbreviations

ARDS: Acute respiratory distress syndrome
BAL: Bronchoalveolar lavage
FITC: Fluorescein isothiocyanate
FR: Fluid resuscitation
GAG: Glycosaminoglycan
MFI: Mean fluorescent intensity
MMP: Matrix metalloproteinase
NFR: No fluid resuscitation
PMN: Polymorphonuclear leukocyte
TIMP: Tissue inhibitors of matrix metalloproteinase
